# The Cost of Excessive Smartphone Use: Guilt Cross the Work-Family Domains

**DOI:** 10.3389/fpsyg.2021.701482

**Published:** 2021-07-20

**Authors:** Yujing Liu, Jing Du, Yuan Li

**Affiliations:** Economics and Management School, Wuhan University, Wuhan, China

**Keywords:** private smartphone use, guilt, family role performance, emotion regulation, ethical compensation

## Abstract

Empirical evidence has accumulated showing that smartphone use at work has the double-edged sword impacts on work-related attitudes and behaviors, but little is known about how its effects transmit and spill over from the workplace to the family domain. Drawing upon compensatory ethics theory, we hypothesize positive associations of employees’ daily private smartphone use at work with their family role performance after work through feeling of guilt. Using an experience sampling methodology, we test our hypotheses in a sample of 101 employees who completed surveys across 10 consecutive workdays. Multilevel path analysis results showed that excessive smartphone use at work triggered experienced guilt, and had a positive indirect effect on family role performance via feeling of guilt. Furthermore, employees with high ability of emotion regulation can be better resolve own painful emotion by engaging in family role performance. Theoretical and practical implications, limitations, and propose future research directions are discussed.

## Introduction

It is obvious that the advent of mobile devices (i.e., smartphones) facilitates widespread and rapid information sharing and communications among employees at anytime, anyplace (Park et al., 2020). When using smartphone for private purposes during worktime, there is also evidence to support its potential recovery effects as a way of micro-break (Rieger et al., 2017). However, private smartphone use at work is indeed regarded as counterproductive work behavior or workplace deviance from the cyber slacking perspective (Derks et al., 2021). Due to violating workplace norms and undermining productivity, such non-work smartphone use behaviors might been seen by oneself and by others as morally discrediting (Klotz and Bolino, 2013). That means employees who use smartphone excessively at work may feel guilty because of the loss of moral credits (Liao et al., 2018). Importantly, knowledge about whether and in what ways those work-related feelings after private smartphone use have ramifications for employees’ life is not clear.

Work and family are not discrete domains, and they are intertwined that what happens in one domain is likely to affect what happens in the other (Chen et al., 2014). From boundary management perspective, Derks et al. (2016) has investigated that daily work-related smartphone use outside of traditional working hour is related to daily family role performance, whether it has the positive or negative effect depends on employees’ levels of segmentation preferences. But we still know little about the impact of private (i.e., non-work relevant) smartphone use in workplace on employees’ family role performance. When employees experience work-related guilt after excessive private smartphone use, those feelings may not leave in the work domain but instead cross over to affect life in the family domain (Thompson et al., 2020). In order to examine the spillover effects of the excessive smartphone during office time on family domain through feeling of guilt, we built a theoretical model on the basis of compensatory ethics theory (Zhong et al., 2010). Drawing upon compensatory ethics theory, an initial unethical behavior may induce subsequent ethical behavior to restore a moral self-image, people will be motivated to show a compensatory reaction rather than a consistency reaction to their previous acts (Mulder and Aquino, 2013). That is, when individuals breach their norms or values, they are more likely to engage in behaviors that affirm core values as compared to behaviors which directly repair the damage caused by the transgression (Tetlock et al., 2000). Therefore, employees can rebuild their own moral credits by displaying higher levels of family role performance in the subsequent context.

**FIGURE 1:**
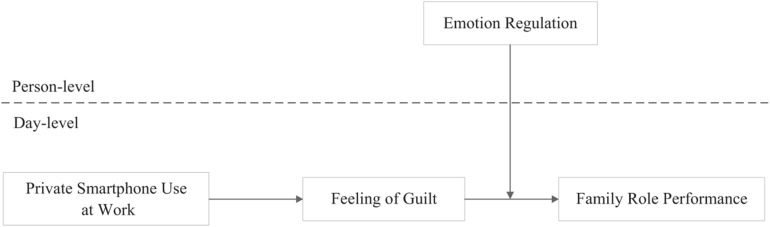
Research conceptual model.

Our theoretical model thus far highlights the role of experienced guilt between daily private smartphone use at work and family role performance. It is also essential to recognize that such effect possibly varies across people depending on their ability of emotion regulation. Emotion regulation consists of an individual’s active attempts to manage own emotional states, which determines the offset of the present emotional response (Koole, 2009). While feeling of guilt means people become aware of the actual and expected violations of internal values, the capacity to regulate emotions within self can signal employees that something is wrong, and stimulate them to take ethical action (Luqman et al., 2020). Hence, we propose that employees with high emotion regulation are more conscious of own negative moral emotions and are driven to improve family role performance than those unskilled at regulating their emotions. The conceptual model as Figure 1 shows.

Our study contributes to the current literature in three ways. First, we establish a theoretical and empirical basis for linking private smartphone use at work to feeling of guilt and associated family behavior. In doing so, we extend the smartphone use literature by providing support for the motivating effects of family role performance according to compensatory ethics theory. Second, the present study helps to understand how the consequences of workplace smartphone use behaviors spill over into lives outside of work. Despite research has suggested that smartphone use is responsible for subsequent exhaustion (Derks et al., 2021), our findings argued that feeling of guilt operate as moral recall, thereby underlie the connection between excessive smartphone use at work and higher performance outside of work. Third, we propose that compared to low levels of emotion regulation, employees with strong emotion-regulation competencies have a greater sense of motivation to modulate their daily emotional fluctuation.

## Theory and Hypotheses

### Private Smartphone Use at Work and Feeling of Guilt

Although the many benefits smartphone use can bring convenience and efficiency to work, employees may be tempted to read private messages by news alert during working hours. Private smartphone use at work in terms of checking and answering for private messages, making private phone calls, and using apps to surfing on the web or track news for entertainment purposes (Dora et al., 2019; Derks et al., 2021). When employees evaluate whether private smartphone use at work was morally wrong, that means they have recognized the moral issue and then make a moral judgment (Rest, 1986). Using smartphone for private purposes violates workplace standards and norms, then along comes the individual negative moral self-appraisal process because they attribute the occurrence of moral transgressions to poor self-control (Tangney, 1990; Liao et al., 2018). Feeling of guilt is a self-conscious moral emotion that arises when employees find that own immoral actions to be emotionally unacceptable (Zhong et al., 2010). Thus, we predict that daily private smartphone use spends personal moral credits that increase individual feeling of guilt.

Hypothesis 1. Daily private smartphone use at work is positively related to daily feeling of guilt.

### Feeling of Guilt and Family Role Performance

Feeling of guilt is generally concern about a particular behavior, which can be painful, and involves a sense of remorse or regret over the bad action (Baumeister et al., 1994; Menesini et al., 2003). As an exemplary moral emotion, the morality of guilt lies in the fact that they can stimulate compensatory prosocial behavior in dyadic social dilemma situations (De Hooge et al., 2011). That is due to individuals in the midst of guilt experience often indicate a nagging focus that think of it over and over, and typically motivates them attempt to offset the bad deed that was done in some way (Tangney et al., 1996). Individuals’ particular role identity can reflect distinct contexts, such as work or family. Specifically, experienced guilt from work domain spills over to the family domain (Carlson et al., 2019), then contributes to individuals’ family role performance.

Family role performance is defined as “the fulfillment of obligations and expectations stemming from the roles associated with participation in the family domain” (Chen et al., 2014, p. 4). Similar to job role performance, family role performance includes getting housework done and facilitating relationships with family members (Chen et al., 2014; Derks et al., 2016). The compensatory ethics theory suggests that initial unethical behaviors reduce moral credits, subsequent moral emotion (e.g., guilt) can encourage employees to make following ethical decisions thereby establishing essential credits (Zhong et al., 2010). Therefore, individuals who felt guilty about excessive smartphone use may fulfill their family responsibilities through dedicating evening hours to family life. Building on the compensatory ethics theory and previous argument, we assume that private smartphone use at work fosters feeling of guilt and undermines own moral image, which triggers family role performance in that are intended to compensate for the guilt-inducing behavior.

Hypothesis 2. Daily feeling of guilt is positively related to daily family role performance.

Hypothesis 3. There are indirect relationships between private smartphone use at work and family role performance through feeling of guilt, such that employees use smartphone for private purposes frequently are more likely to experience guilt and thereby engage in more family role performance.

### Emotion Regulation as Moderating Variable

Individual emotions are not so irresistible as they are generally portrayed, people are much more flexible in dealing with own emotional response. Emotion regulation refers to the ability of people to regulate and control own emotional dynamics (Wong and Law, 2002). The effects of emotion regulation can be observed across multiple modalities of emotional responding, like physiology and behavior (Koole, 2009). Employees with high emotion regulation, that is emotional maturity, are more sensitive to feelings and emotions of themselves. Meanwhile, they should be more able to modulate their behavioral response tendencies after experienced an emotion (Jiang et al., 2013; Gabriel et al., 2020). In other words, employees can be better control own negative emotions and better control over own lives by the capability of emotion regulation (Wong and Law, 2002).

When employees experienced guilt in worktime, emotion regulation determines how easily they can leave such uncomfortable emotional state. Emotion regulation does not always consist of overriding one’s spontaneous emotion, individual may decrease negative emotions by direct attention away from stimuli that could trigger unwanted emotions (Koole, 2009). According to compensatory ethics theory, feeling of guilt may motivate individual to engage in reparative actions as a way to counteract the threatened moral self-concept (Liao et al., 2018). In such case, employees high in emotion regulation are more aware of emotional changes and more likely to feel responsible to resolve prior wrongdoings. When they return home from work, engaging in family role performance naturally become their moral cleansing way and prosocial compensation behavior to relieve moral distress (Gino et al., 2015). Therefore, we propose that feeling of guilt will have a stronger relationship with family role performance among those with high emotion regulation but a weaker relationship among those with low emotion regulation.

Hypothesis 4. Emotion regulation at person-level moderates the day-level relationship between feeling of guilt and family role performance, such that employees with high (low) emotion regulation will be more (less) likely to be involved in family activities.

Combined, our hypotheses (H1–H4) imply that the strength of the indirect relationships between private smartphone use at work and family role performance via feeling of guilt may differ by levels of emotion regulation. The ability of emotion regulation helps employees to monitor their emotional fluctuation and endeavor to maintain a favorable emotional state by balancing own moral and immoral behaviors (Zhong et al., 2010). Taken together, we suggest that the indirect effects of private smartphone use at work on family role performance are stronger for employees with high emotion regulation, compared to employees with low emotion regulation.

Hypothesis 5. The day-level indirect effects of private smartphone use at work on family role performance through feeling of guilt are stronger (weaker) for employees with higher (lower) levels of emotion regulation.

## Materials and Methods

### Participants and Procedure

We recruited participants from a wide variety of organizations with the snowball sampling technique, that is the initial participants were invited from an MBA program, and then they recommended to others who might be interested in this study (Puranik et al., 2021). The enrolled participants need to live with at least one family member, in order to have opportunity of displaying family role performance. Before beginning data collection, we sent all participants an announcement explaining the study procedure (a general survey and two daily surveys for 10 workdays) and assuring the voluntariness of participation and the confidentiality of their responses.

We sent messages to participants with website links containing corresponding online questionnaires. In the general questionnaire, participants reported emotion regulation and demographic characteristics such as gender, age, and organizational tenure. During the daily portion of the study, we sent two short diary questionnaires every day for 10 consecutive workdays. Specifically, participants received the first survey before the end of workday (Time1, 6:00 p.m.) to assess private smartphone use at work and feeling of guilt. On average, it was completed at 6:37 p.m. The second survey was sent at night (Time2, 9:00 p.m.) and captured participants’ behavior since arriving at home. It contained the scale to measure family role performance, and was completed on average, at 9:25 p.m. Given that participants especially in diary designs prefer single item and are more motivated to provide accurate responses (Aarntzen et al., 2019), we chose the short measurement as possible. All survey items were self-reported, because we mainly focus on employees’ experience and subsequent response. Research also showed that when it comes to such state variables, it is acceptable for using the same-source data (Puranik et al., 2021).

Overall, our sample comprised 105 respondents that were occupied with various managerial, administrative and technical positions in own organizations. We removed four participants who contributed fewer than three complete daily data points (Rosen et al., 2019). Our final data thus included 944 matched daily data from 101 participants (average of 9.3 days per person). Their average age was 32.68 years (*SD* = 4.97), and 53% were female. Average organizational tenure was 9.97 years (*SD* = 5.04).

### Day-Level Measures

Daily private smartphone use at work was assessed by asking the participants to rate the frequency of private smartphone-use behavior (Kim et al., 2018). The adapted item asked participants to recall their smartphone-use taken for informal purposes during worktime and then rate how often they used smartphone for private (1 = never to 5 = very frequently).

Feeling of guilt was measured with the one-item work-family guilt scale developed by Aarntzen et al. (2019). We explicitly added a reference to time (today) in our measure. The adapted item was: “When you think about how you used smartphone for private today, to what extent do you feel guilty toward yourself. Today I feel …” with a scale ranging from 1 = not at all guilty to 5 = very guilty.

Family role performance was evaluated by using the four-item scale developed by Chen et al. (2014). Participants were asked to rate the extent to which they fulfill what is expected of them in terms of their daily family role life. Example items were: “Today I complete household responsibilities,” and “Today I provide emotional support to my family members.” All items were rated on five-point Likert scale (1 = do not fulfill expectations at all to 5 = fulfill expectations completely). Average reliability over all 10 research days was α = 0.95.

### Person-Level Measure

Emotion regulation was measured with the four-item ability emotional intelligence scale developed by Wong and Law (2002). Example items were: “I am able to control my temper and handle difficulties rationally,” and “I can always calm down quickly when I am very angry.” All items were rated on five-point Likert scale (1 = totally disagree to 5 = totally agree). Cronbach’s α of the scale was 0.87.

### Analytical Strategy

Our research design resulted in multilevel model with daily responses at the day-level (*n* = 944 study occasions) and individual participants at the person-level (*N* = 101 participants). To consider the day-level and person-level variances when testing the theoretical model shown in Figure 1, we conducted multilevel path analysis with Mplus 7.4 (Muthén and Muthén, 1998–2017). The variables at day-level (daily private smartphone use at work, daily feeling of guilt and daily family role performance) were centered to the group-mean and person-level variable (emotion regulation) was centered to the grand-mean (Aguinis et al., 2013; Sherf et al., 2019). In addition, we followed Preacher and Selig’s (2012) suggestion to test the significance of the indirect effects by parametric bootstrapping technique, and estimated confidence intervals based on Monte Carlo simulations with 20,000 replications using the open-source software R (Preacher et al., 2010).

## Results

### Descriptive Statistics

Table 1 reports the means, standard deviations, and correlations among the study variables. For each daily variable, the proportion of within-person variance was calculated as within-person variance divided by the sum of within-person variance and between-person variance (Ilies et al., 2017). Results showed that the proportion of within-person variance in private smartphone use at work is 55.09%, the proportion of within-person variance in feeling of guilt is 56.65%, and the proportion of within-person variance in family role performance is 53.58%. That means there was sufficient within-person variance for daily variables in the current study (Ilies et al., 2017).

**TABLE 1:** Descriptive statistics and correlations among study variables.

Variables	Mean	SD_*w*_	SD_*b*_	1	2	3	4	5	6	7
1. Private smartphone use	2.29	0.97	0.70	−	0.67**	0.18	−0.21*	–0.09	–0.11	−0.20*
2. Feeling of guilt	2.06	0.82	0.58	0.62**	−	0.07	–0.16	0.03	0.06	−0.22*
3. Family role performance	3.26	0.73	0.53	0.14**	0.08*	−	0.14	–0.17	−0.22*	–0.08
4. Gender	1.52	−	0.50	−0.15**	−0.12**	0.10**	−	−0.27**	−0.26**	−0.22*
5. Age	32.68	−	4.97	–0.05	0.03	−0.12**	−	−	0.91**	–0.11
6. Organizational tenure	9.97	−	5.04	−0.07*	0.06	−0.16**	−	−	−	–0.02
7. Emotion regulation	3.39	−	0.57	−0.13**	−0.15**	–0.04	−	−	−	−

Within-person correlations are shown below the diagonal (n = 944). Between-person correlations are shown above the diagonal, with daily variables averaged across the study days (N = 101). _*w*_ = Within-person. _*b*_ = Between-person. *p < 0.05, **p < 0.01.

### Hypotheses Testing

Table 2 presents the results from the multilevel path analysis that estimated all path coefficients. Private smartphone use at work was positively related to increased feeling of guilt (γ = 0.48, *p* < 0.001), supporting Hypothesis 1. Feeling of guilt was positively related to family role performance (γ = 0.11, *p* < 0.05), Hypothesis 2 was supported. Individuals’ feeling of guilt was further hypothesized to influence the relationship between private smartphone use at work and family role performance. Results showed that the indirect effect of private smartphone use at work on family role performance via feeling of guilt was 0.05 with a 95% bias-corrected bootstrap confidence interval (CI) of 0.01 to 0.10. Because the 95% CI did not include zero, Hypothesis 3 was supported.

**TABLE 2:** Unstandardized coefficients of the multilevel model.

	Feeling of guilt		Family role performance			
	Estimate	SE	Estimate	SE	Estimate	SE
Intercept	2.06***	0.06	3.25***	0.05	3.25***	0.05
Gender	–0.19	0.12	0.09	0.11	0.07	0.12
Age	–0.02	0.03	0.02	0.03	0.02	0.03
Organizational tenure	0.02	0.03	–0.04	0.04	–0.04	0.04
Private smartphone use at work	0.48***	0.04				
Feeling of guilt			0.11*	0.05	0.07*	0.04
Emotion regulation					–0.03	0.11
Feeling of guilt × emotion regulation					0.14*	0.06
Pseudo-R^2^	0.55		0.52		0.53	

Data points = 944 (respondents = 101, days = 10). SE = standard error. Pseudo-R^2^ indicates percentage of the total variance in the dependent variable accounted by all predictor variables based on formulas suggested by Sherf et al. (2019). *p < 0.05, ***p < 0.001.

**FIGURE 2:**
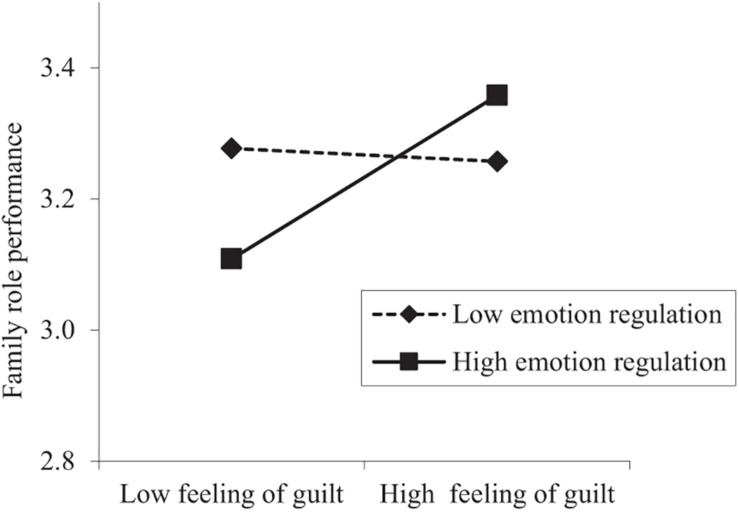
Moderation effect of emotion regulation on the relationship between daily feeling of guilt and family role performance.

We tested the cross-level moderation effect of emotion regulation on the day-level relationship between feeling of guilt and family role performance. The results in Table 2 showed that emotion regulation was positively associated with the random slopes between feeling of guilt and family role performance (γ = 0.14, *p* < 0.05). We plotted interaction effects testing the predictor at conditional values of emotion regulation (1 SD above and below the mean), as shown in Figure 2. We also conducted a simple slope analysis as recommended by Preacher et al. (2006). The results showed that the positive day-level relationship between feeling of guilt and family role performance existed for those who had high levels (+1 SD) of emotion regulation (γ = 0.16, SE = 0.05, *p* < 0.01) but not for those who had low levels (−1 SD) of emotion regulation (γ = −0.02, SE = 0.05, *p* = 0.74). Thus, Hypothesis 4 was supported.

Last, we tested whether the estimated indirect effect of private smartphone use at work on family role performance via feeling of guilt differed at lower (*−*1 SD) and higher (+1 SD) levels of emotion regulation. The results showed that private smartphone use had a significant indirect effect of 0.06 (*p* < 0.05) under high emotion regulation levels, and this effect was not significant under low emotion regulation levels (*−*0.01, *p* = 0.69). Moreover, the estimate of the difference between the two indirect effects was 0.07 (95% CI [0.01, 0.13]). Therefore, emotion regulation significantly moderated the indirect relationship between daily private smartphone use at work and family role performance via feeling of guilt, providing support for Hypothesis 5. The results for hypotheses testing are summarized in Table 3.

**TABLE 3:** Results for hypotheses testing.

Hypotheses	Results
Hypothesis 1. Daily private smartphone use at work is positively related to daily feeling of guilt.	Acceptance
Hypothesis 2. Daily feeling of guilt is positively related to daily family role performance.	Acceptance
Hypothesis 3. There are indirect relationships between private smartphone use at work and family role performance through feeling of guilt, such that employees use smartphone for private purposes frequently are more likely to experience guilt and thereby engage in more family role performance.	Acceptance
Hypothesis 4. Emotion regulation at person-level moderates the day-level relationship between feeling of guilt and family role performance, such that employees with high (low) emotion regulation will be more (less) likely to be involved in family activities.	Acceptance
Hypothesis 5. The day-level indirect effects of private smartphone use at work on family role performance through feeling of guilt are stronger (weaker) for employees with higher (lower) levels of emotion regulation.	Acceptance

## Discussion

Smartphone is a mobile device with extended features to browse the Internet, play games and have access to social media, its flexibility can help employees to fulfill own responsibilities from both work and family simultaneously (Towers et al., 2006), but also can provide opportunity for them to cyberslacking. Although previous research has showed the effects of work-related smartphone use during off-job hours on family role performance (Derks et al., 2016), the potential impacts of family domains from private smartphone use during worktime have received less attention. According to compensatory ethics theory (Zhong et al., 2010), we developed and tested a model spanning work and family domains to explain how and when excessive private smartphone use at work affects employees’ feeling and family performance. Findings from an experience sampling study over 2-week period revealed that using too much smartphone for private purposes triggered individual feeling of guilt, and in turn had motivating effects on their family role performance. Moreover, these effects were constrained by personal ability of emotion regulation, such that employees engaged in family as the reparative response only when they are able to manage own emotional states.

### Theoretical Contributions

Our central contribution is to advance the smartphone use literature, by shedding light on the non-work-related smartphone use at work has remarkable influence to family role performance via feeling of guilt. The adoption of smartphone has significantly blurred the boundary between work and family domains, which result in great changes in the way employees complete work and family interactions (Olson-Buchanan and Boswell, 2006). Previous studies have demonstrated the negative effects of excessive private smartphone use on personal mental resources (Derks et al., 2021), the promotive implications for the family domain have received less research attention. Consistent with the compensatory ethics theory (Zhong et al., 2010), our study explores that moral emotion arisen during worktime can lead to the compensatory effect, and then spill over into own family life. When employees were perceived guilt for contravening work rules, they are more likely to apologize for their unethical action and make amends for moral credits deficit (Ketelaar and Au, 2003). As a result, employees can make up for moral credit loss arising from excessive smartphones use in workplace by taking on family responsibilities.

In addition, the present study contributes to our understanding of the cross-domain possibility in which moral compensatory behavior will occur. Yam et al. (2017) found that moral license-generating and its subsequent behavior need not always be in the same domain. Similarly, after recognizing guilt feeling at work, employees’ ensuing ethical compensatory behavior will not be limited to the workplace. A job that has the opportunity to spend a lot of time on private smartphone use just shows employees may be under the low workload, that implies they do not require to perform remedial actions by working overtime to complete the work. Our study empirically demonstrates the mechanism through which employees’ on-the-job excessive smartphone use can significantly influence their behaviors outside of work, thereby stimulating interaction with family members as the continuity of ethical memory. By adopting compensatory ethics theory to explain the potential impact of excessive smartphone use on work-family interaction, the present study provides empirical evidence to support the argument that moral compensatory behavior can be existed in cross-domain.

Finally, our results extend previous research by suggesting the cross-level moderating role of emotion regulation in moral compensation process (Ding et al., 2016). Negative moral emotions associated with an action may lead individual to pay attention to personal moral credentials (Zhong et al., 2010). When employees make a relatively unethical decision, employees who regulate their emotions effectively are driven to act more ethically on next ethical choice. In other words, emotion regulation can facilitate the direct effect from experienced guilt by excessive smartphone use to family performance. The result of this study indicates that the indirect influence of private smartphone use at work on family role performance through feeling of guilt is stronger when the level of employee’s emotion regulation is higher, but is weaker when the level of employee’s emotion regulation is lower. This finding also answers the call for further investigation about the role of emotion regulation in social interactions (Wong and Law, 2002).

### Practical Implications

Our findings provide serval implications for organizations and employees. Given the widespread application of smartphone, completely banning smartphone use for private purposes during worktime might be the unrealizable solution (Derks et al., 2021). Our study demonstrates that employees engaging in private smartphone use yields negative emotional response for themselves as well. To relieve such emotional experiences of immorality, they must perform other ethical behaviors at a level above and beyond what they normally do (Liao et al., 2018). Consequently, organizations may benefit from finding ways to induce employees to proactively helping colleague or other pro-organizational behaviors within work domain. In doing so, employees could similarly regulate themselves from negative moral emotions and potentially contribute to the whole team performance. Furthermore, considering that higher ability of emotion regulation is characterized by greater self-reflexivity and more profound awareness of own emotions (Koole, 2009), which is a form of self-regulation (Gross, 2002). It is necessary to enhance employees’ emotion regulation competency through providing reflective assignments on attentional deployment (Hülsheger et al., 2015).

### Limitations and Future Research Directions

The present study has some limitations that should be acknowledged. First, we used one single item to measure private smartphone use at work and feeling of guilt, that is because we hope to reduce the participants’ burden of daily questions and to optimize overall response rates (Aarntzen et al., 2019). Although prior research has proven that single item measurement is reliable and valid for unidimensional constructs such as happiness and life satisfaction (Abdel-Khalek, 2006; Cheung and Lucas, 2014; Robins et al., 2016), as well as has been used in questionnaire survey before (Smith et al., 2007; Du and Choi, 2010), it is still necessary to examine whether there is difference between single-item measurement and multiple-items measurement about these two variables. Therefore, we conducted a supplementary study that was collected from 28 part-time MBA students for 5 workdays. Private smartphone use at work was assessed with four-item scale adapted by Derks et al. (2021). An example items is “Today, I felt obligated to answer private messages on my smartphone.” Feeling of guilt was measured with the four-item scale adapted by Bonner et al. (2017). An example items is “When thinking about how I used smartphone for private today, I feel dissatisfied with self.” The measurement of family role performance is consistent with the diary questionnaires we mentioned above.

The multilevel path analysis showed that private smartphone use at work was positively related to feeling of guilt (γ = 0.17, *p* < 0.001), feeling of guilt was positively related to family role performance (γ = 0.24, *p* < 0.05). The indirect effect of private smartphone on family role performance via feeling of guilt was 0.04 (95% CI [0.01, 0.08]). Meanwhile, we analyzed the results of using single item measurement. There is also a positive relationship between private smartphone use at work and feeling of guilt (γ = 0.39, *p* < 0.01), and a positive relationship between feeling of guilt and family role performance (γ = 0.16, *p* < 0.001). We observed positive indirect effects of private smartphone on family role performance via feeling of guilt (indirect effect = 0.04, 95% CI [0.01, 0.08]). It follows that the relevant hypotheses were all tested regardless of whether multiple or single items were used.

Second, experimental designs, rather than cross sectional study, can provide the strongest foundation for making causal inferences (Clark et al., 2019). To establish causality among study variables, we invite scholars to build further on our findings by manipulating experiments with random assignments. Alternatively, a cross-lagged survey design could allow for stronger causal inferences. Third, our participants were limited and we only conducted the study in China. Future research should examine to what extent our conclusions could be generalized to various cultural contexts.

In addition, we explored the moderating effects of emotion regulation on the relationship of experienced guilt induced by excessive smartphone use with its spillover benefits for family. Future research could take into account the motivation behind private smartphone use at work when investigating the consequence of excessive smartphone use. For example, self-determination theory proposed that the motivation for displaying a behavior is differentiated ranging from controlled motivation to autonomous motivation (Ohly and Latour, 2014). If employees have an autonomous motivation to use smartphone for private purposes, like detaching from the too complex work, these behaviors may weaken feeling of guilt because of the satisfaction of their basic psychological needs.

Finally, individuals differ in the extent to which they allow both domains (family and work) to be integrated, which depend on their own preferences and might influence the effect of cross-domain smartphone usage in the shape of guilt and family performance role (Ashforth et al., 2000). For example, previous research results indicated that stronger work role identification was related to higher work-family boundaries integration tendency and cross-domain smartphone use (Olson-Buchanan and Boswell, 2006). Moreover, the expectations and norms for using smartphone has also been identified as important factors for predicting cross-domain smartphone use (Bittman et al., 2009; Matusik and Mickel, 2011). Future research should consider relevant contextual factor, that is individuals’ boundary management preferences and the expectations and norms for using smartphone. Such further examination would provide more nuances about the relationship between private smartphone use in workplace and family role performance.

## Data Availability Statement

The raw data supporting the conclusions of this article will be made available by the authors, without undue reservation, to any qualified researcher.

## Ethics Statement

The studies involving human participants were reviewed and approved by the Ethics Committee of Wuhan University. The participants provided their written informed consent to participate in this study.

## Author Contributions

All authors contributed conception and design of the study, collected the database, performed the statistical analysis, wrote the first draft and sections of the manuscript, and contributed to manuscript revision, read and approved the submitted version.

## Conflict of Interest

The authors declare that the research was conducted in the absence of any commercial or financial relationships that could be construed as a potential conflict of interest.

## References

[B1] Aarntzen L. Derks B. Steenbergen E. V. Ryan M. Tanja V. (2019). Work-family guilt as a straightjacket. An interview and diary study on consequences of mothers’ work-family guilt. *J. Vocat. Behav.* 115:103336. 10.1016/j.jvb.2019.103336

[B2] Abdel-Khalek A. M. (2006). Measuring happiness with a single-item scale. *Soc. Behav. Pers.* 34 139–150. 10.2224/sbp.2006.34.2.139

[B3] Aguinis H. Gottfredson R. K. Culpepper A. (2013). Best-practice recommendations for estimating cross-level interaction effects using multilevel modeling. *J. Manage.* 39 1–39.

[B4] Ashforth B. E. Kreiner G. E. Fugate M. (2000). All in a day’s work: boundaries and micro role transitions. *Acad. Manage. Rev.* 25 472–491. 10.2307/259305

[B5] Baumeister R. F. Stillwell A. M. Heatherton T. F. (1994). Guilt: an interpersonal approach. *Psychol. Bull.* 115 243–267. 10.1037/0033-2909.115.2.243 8165271

[B6] Bittman M. Brown J. E. Wajcman J. (2009). The mobile phone, perpetual contact and time pressure. *Work Employ. Soc.* 23 673–691. 10.1177/0950017009344910

[B7] Bonner J. M. Greenbaum R. L. Quade M. J. (2017). Employee unethical behavior to shame as an indicator of self-image threat and exemplification as a form of self-image protection: the exacerbating role of supervisor bottom-line mentality. *J. Appl. Psychol.* 102 1203–1221. 10.1037/apl0000222 28383944

[B8] Carlson D. S. Thompson M. J. Kacmar K. M. (2019). Double crossed: the spillover and crossover effects of work demands on work outcomes through the family. *J. Appl. Psychol.* 104 214–228. 10.1037/apl0000348 30179020

[B9] Chen Y. Shaffer M. Westman M. Chen S. Lazarova M. Reiche S. (2014). Family role performance: scale development and validation. *Appl. Psychol.* 63 190–218. 10.1111/apps.12005

[B10] Cheung F. Lucas R. E. (2014). Assessing the validity of single-item life satisfaction measures: results from three large samples. *Qual. Life Res.* 23 2809–2818. 10.1007/s11136-014-0726-4 24890827 PMC4221492

[B11] Clark M. A. Robertson M. M. Young S. (2019). “I feel your pain”: a critical review of organizational research on empathy. *J. Organ. Behav.* 40 166–192. 10.1002/job.2348

[B12] De Hooge I. E. Nelissen R. M. A. Breugelmans S. M. Zeelenberg M. (2011). What is moral about guilt? acting “prosocially” at the disadvantage of others. *J. Pers. Soc. Psychol.* 100 462–473. 10.1037/a0021459 21244173

[B13] Derks D. Bakker A. B. Gorgievski M. (2021). Private smartphone use during worktime: a diary study on the unexplored costs of integrating the work and family domains. *Comput. Hum. Behav.* 114:106530. 10.1016/j.chb.2020.106530

[B14] Derks D. Bakker A. B. Peters P. Wingerden P. V. (2016). Work-related smartphone use, work-family conflict and family role performance: the role of segmentation preference. *Hum. Relat.* 69 1045–1068. 10.1177/0018726715601890

[B15] Ding W. Xie R. Sun B. Li W. J. Wang D. Zhen R. (2016). Why does the “sinner” act prosocially? the mediating role of guilt and the moderating role of moral identity in motivating moral cleansing. *Front. Psychol.* 7:1317. 10.3389/fpsyg.2016.01317 27660617 PMC5014871

[B16] Dora J. Hooff M. Geurts S. Hooftman W. E. Kompier M. (2019). Characterizing work-related smartphone use at home and private smartphone use at work using latent class analysis. *Occup. Health Sci.* 3 187–203. 10.1007/s41542-019-00040-6

[B17] Du J. Choi J. N. (2010). Pay for performance in emerging markets: insights from China. *J. Int. Bus. Stud.* 41 671–689. 10.1057/jibs.2009.40

[B18] Gabriel A. S. Koopman J. Rosen C. C. Arnold J. D. Hochwarter W. A. (2020). Are coworkers getting into the act? An examination of emotion regulation in coworker exchanges. *J. Appl. Psychol.* 105 907–929. 10.1037/apl0000473 31789551

[B19] Gino F. Kouchaki M. Galinsky A. D. (2015). The moral virtue of authenticity: how inauthenticity produces feelings of immorality and impurity. *Psychol. Sci.* 26 983–996. 10.1177/0956797615575277 25963614

[B20] Gross J. J. (2002). Emotion regulation: affective, cognitive, and social consequences. *Psychophysiology* 39 281–291. 10.1017/s0048577201393198 12212647

[B21] Hülsheger U. R. Lang J. W. B. Schewe A. F. Zijlstra F. R. H. (2015). When regulating emotions at work pays off: a diary and an intervention study on emotion regulation and customer tips in service jobs. *J. Appl. Psychol.* 100 263–277. 10.1037/a0038229 25384203

[B22] Ilies R. Liu X. Y. Liu Y. Zheng X. (2017). Why do employees have better family lives when they are highly engaged at work? *J. Appl. Psychol.* 102 956–970. 10.1037/apl0000211 28277725

[B23] Jiang J. Y. Zhang X. Tjosvold D. (2013). Emotion regulation as a boundary condition of the relationship between team conflict and performance: a multi-level examination. *J. Organ. Behav.* 34 714–734. 10.1002/job.1834

[B24] Ketelaar T. Au W. T. (2003). The effects of guilt on the behaviour of uncooperative individuals in repeated social bargaining games: an affect-as-information interpretation of the role of emotion in social interaction. *Cogn. Emot.* 17 429–453. 10.1080/02699930143000662 29715746

[B25] Kim S. Park Y. Headrick L. (2018). Daily micro-breaks and job performance: general work engagement as a cross-level moderator. *J. Appl. Psychol.* 103 772–786. 10.1037/apl0000308 29595289

[B26] Klotz A. C. Bolino M. C. (2013). Citizenship and counterproductive work behavior: a moral licensing view. *Acad. Manage. Rev.* 38 292–306. 10.5465/amr.2011.0109

[B27] Koole S. L. (2009). The psychology of emotion regulation: an integrative review. *Cogn. Emot.* 23 4–41. 10.1080/02699930802619031

[B28] Liao Z. Yam K. C. Johnson R. Liu W. Song Z. (2018). Cleansing my abuse: a reparative response model of perpetrating abusive supervisor behavior. *J. Appl. Psychol.* 103 1039–1056. 10.1037/apl0000319 29722999

[B29] Luqman A. Masood A. Weng Q. D. Ali A. Rasheed M. I. (2020). Linking excessive SNS use, technological friction, strain, and discontinuance: the moderating role of guilt. *Inf. Syst. Manage.* 37 94–112. 10.1080/10580530.2020.1732527

[B30] Matusik S. F. Mickel A. E. (2011). Embracing or embattled by converged mobile devices? users’ experiences with a contemporary connectivity technology. *Hum. Relat.* 64 1001–1030. 10.1177/0018726711405552

[B31] Menesini E. Sanchez V. Fonzi A. Ortega R. Costabile A. Feudo G. L. (2003). Moral emotions and bullying: a cross-national comparison of differences between bullies, victims and outsiders. *Aggress. Behav.* 29 515–530. 10.1002/ab.10060

[B32] Mulder L. B. Aquino K. (2013). The role of moral identity in the aftermath of dishonesty. *Organ. Behav. Hum. Dec.* 121 219–230. 10.1016/j.obhdp.2013.03.005

[B33] Muthén Muthén (1998–2017). *Mplus User’s Guide*, 8th Edn. Los Angeles, CA: Muthén and Muthén.

[B34] Ohly S. Latour A. (2014). Work-related smartphone use and well-being in the evening: the role of autonomous and controlled motivation. *J. Pers. Psychol.* 13 174–183.

[B35] Olson-Buchanan J. Boswell W. (2006). Blurring boundaries: correlates of integration and segmentation between work and nonwork. *J. Vocat. Behav.* 68 432–445. 10.1016/j.jvb.2005.10.006

[B36] Park Y. A. Liu Y. Headrick L. (2020). When work is wanted after hours: testing weekly stress of information communication technology demands using boundary theory. *J. Organ. Behav.* 41 518–534. 10.1002/job.2461

[B37] Preacher K. J. Curran P. J. Bauer D. J. (2006). Computational tools for probing interactions in multiple linear regression, multilevel modeling, and latent curve analysis. *J. Educ. Behav. Stat.* 31 437–448. 10.3102/10769986031004437

[B38] Preacher K. J. Selig J. P. (2012). Advantages of Monte Carlo confidence intervals for indirect effects. *Commun. Methods Meas.* 6 77–98. 10.1080/19312458.2012.679848

[B39] Preacher K. J. Zyphur M. J. Zhang Z. (2010). A general multilevel SEM framework for assessing multilevel mediation. *Psychol. Methods* 15 209–233. 10.1037/a0020141 20822249

[B40] Puranik H. Koopman J. Vough H. C. (2021). Excuse me, do you have a minute? an exploration of the dark- and bright-side effects of daily work interruptions for employee well-being. *J. Appl. Psychol.* 10.1037/apl0000875 [Epub ahead of print]. 33600194

[B41] Rest J. R. (1986). *Moral Development: Advances in Research and Theory.* New York, NY: Praeger.

[B42] Rieger D. Hefner D. Vorderer P. (2017). Mobile recovery? The impact of smartphone use on recovery experiences in waiting situations. *Mob. Media Commun.* 5 161–177. 10.1177/2050157917691556

[B43] Robins R. W. Hendin H. M. Trzesniewski K. H. (2016). Measuring global self-esteem: construct validation of a single-item measure and the Rosenberg self-esteem scale. *Pers. Soc. Psychol. Bull.* 27 151–161. 10.1177/0146167201272002

[B44] Rosen C. C. Simon L. S. Gajendran R. S. Johnson R. E. Lee H. W. Lin J. (2019). Boxed in by your inbox: implications of daily email demands for managers’ leadership behaviors. *J. Appl. Psychol.* 104 19–33. 10.1037/apl0000343 30221954

[B45] Sherf E. N. Vijaya V. Gajendran R. S. (2019). Too busy to be fair? the effect of workload and rewards on managers’ justice rule adherence. *Acad. Manage. J.* 62 469–502. 10.5465/amj.2016.1061

[B46] Smith E. R. Seger C. R. Mackie D. M. (2007). Can emotions be truly group level? Evidence regarding four conceptual criteria. *J. Pers. Soc. Psychol.* 93 431–446. 10.1037/0022-3514.93.3.431 17723058

[B47] Tangney J. P. (1990). Assessing individual differences in proneness to shame and guilt: development of the self-conscious affect and attribution inventory. *J. Pers. Soc. Psychol.* 59 102–111. 10.1037/0022-3514.59.1.102 2213483

[B48] Tangney J. P. Miller R. S. Flicker L. Barlow D. H. (1996). Are shame, guilt, and embarrassment distinct emotions? *J. Pers. Soc. Psychol.* 70 1256–1269. 10.1037/0022-3514.70.6.1256 8667166

[B49] Tetlock P. E. Kristel O. V. Elson S. B. Green M. C. Lerner J. S. (2000). The psychology of the unthinkable: taboo trade-offs, forbidden base rates, and heretical counterfactuals. *J. Pers. Soc. Psychol.* 78 853–870. 10.1037/0022-3514.78.5.853 10821194

[B50] Thompson M. J. Carlson D. S. Kacmar K. M. Vogel R. M. (2020). The cost of being ignored: emotional exhaustion in the work and family domains. *J. Appl. Psychol.* 105 186–195. 10.1037/apl0000433 31282700

[B51] Towers I. Duxbury L. Higgins C. Thomas J. (2006). Time thieves and space invaders: technology, work and the organization. *J. Organ. Change Manage.* 19 593–618. 10.1108/09534810610686076

[B52] Wong C. S. Law K. S. (2002). The effects of leader and follower emotional intelligence on performance and attitude: an exploratory study. *Leadersh. Q.* 13 243–274. 10.1016/s1048-9843(02)00099-1

[B53] Yam K. C. Klotz A. C. He W. Reynolds S. J. (2017). From good soldiers to psychologically entitled: examining when and why citizenship behavior leads to deviance. *Acad. Manage. J.* 60 373–396. 10.5465/amj.2014.0234

[B54] Zhong C. B. Ku G. Lount R. B. Murnighan J. K. (2010). Compensatory ethics. *J. Bus. Ethics* 92 323–339. 10.1007/s10551-009-0161-6

